# Validation and extension of an empirical Bayes method for SNP calling on Affymetrix microarrays

**DOI:** 10.1186/gb-2008-9-4-r63

**Published:** 2008-04-03

**Authors:** Shin Lin, Benilton Carvalho, David J Cutler, Dan E Arking, Aravinda Chakravarti, Rafael A Irizarry

**Affiliations:** 1 McKusick-Nathans Institute of Genetic Medicine, Johns Hopkins University School of Medicine, N. Broadway, Baltimore, MD 21205, USA; 2 Department of Biostatistics, Johns Hopkins Bloomberg School of Public Health, North Wolfe St. E3035, Baltimore, MD 21205, USA

## Abstract

Multiple algorithms have been developed for the purpose of calling single nucleotide polymorphisms (SNPs) from Affymetrix microarrays. We extend and validate the algorithm CRLMM, which incorporates HapMap information within an empirical Bayes framework. We find CRLMM to be more accurate than the Affymetrix default programs (BRLMM and Birdseed). Also, we tie our call confidence metric to percent accuracy. We intend that our validation datasets and methods, refered to as SNPaffycomp, serve as standard benchmarks for future SNP calling algorithms.

## Abstract 2

Extended and validated CRLMM is shown to be more accurate than the Affymetrix default programs, and datasets and methods for validation are presented that can serve as standard benchmarks by which future SNP chip calling algorithms can be measured.

## Background

Genome-wide association studies hold great promise in discovering genes underlying complex, heritable disorders for which less powerful study designs have failed in the past [1-3]. Much effort spanning academia and industry and across multiple disciplines has already been invested in making this type of study a reality, with the most recent and largest effort being the Human HapMap Project [4].

Single nucleotide polymorphism (SNP) microarrays represent a key technology allowing for the high throughput genotyping necessary to assess genome-wide variation and conduct association studies [5-9]. Over the years, Affymetrix has introduced SNP microarrays of ever increasing density. The GeneChip^® ^Human Mapping 100K and 500K arrays are beginning to be widely used in association studies, and the 6.0 array with >900,000 SNPs has recently been introduced. At these genotype densities, association studies are theoretically well-powered to detect variants of small phenotypic effect in samples involving hundreds to thousands of subjects [10], and indeed, a number of such successes have recently been reported [11-16].

Practically though, the use of SNP microarrays in association studies has not been entirely straightforward. Genotyping errors, even at a low rate, are known to produce large numbers of putative disease loci, which upon further investigation are found to be false positives. Work by Mitchell and colleagues [17] suggests a per single SNP rate of 0.5% as a maximal threshold for error, particularly for family-based tests. Arriving short of a dataset with such a low rate of error is not so much a failure of the microarray platform *per se *but rather the inadequacy of current SNP calling programs to extract the greatest information from the raw data and, more importantly, to quantify SNP quality, so that unreliable SNPs may be eliminated from further analysis.

In general, genotyping algorithms make a call (AA, AB, or BB) for a SNP of each sample assuming diploids. Typically, a confidence measure is also attached to each genotype call. The user can then choose a level of confidence required for a call to be dropped. One of the first algorithms designed for calling SNPs was Adaptive Background genotype Calling Scheme (ABACUS) [18]. Originally developed for use with the Variation Detection Array (a prototype of current SNP arrays), the method fits Gaussian models using probe intensities associated with a particular SNP of a single chip. A shortcoming of the program is that it has a propensity to drop heterozygous calls. Later, Affymetrix developed Modified Partitioning Around Medoids (MPAM) [19] as the default algorithm for analysis of the 10K chip. The program aggregates the probe intensities across all chips of a SNP, clusters the result, and assigns a genotype call to each cluster. The method does not perform well when the number of chips input into the program is of moderate size and for SNPs with a low minor allele frequency. The latter became a more serious problem when a larger number of such SNPs appeared on the 100K and 500K arrays. As a result, Affymetrix developed the Dynamic Model (DM) [20], a method principally based upon ABACUS. Though unaffected by small sample size and low minor allele frequency, DM, like ABACUS, is prone to drop heterozygous calls.

Recently, Rabbee and Speed [21] developed a method, the Robust Linear Model with Mahalanobis Distance Classifier (RLMM), along the lines of MPAM's basic framework of assigning calls based on clusters but with several novel features. A large proportion of SNPs on Affymetrix chips (>95%) were genotyped in Centre d'Etude du Polymorphisme Humain samples as part of the HapMap Project [4], and these data are used as a training set to pre-define the clusters in RLMM. For SNPs in which certain clusters remain ill defined due to low minor allele frequency, a regression strategy is used to infer cluster characteristics. Though this new algorithm makes calls with markedly greater accuracy than DM, it is not robust to variability in procedures used by different laboratories [21].

Affymetrix has recently introduced a new algorithm, Bayesian Robust Linear Model with Mahalanobis Distance Classifier (BRLMM) [22], which is the default program for Affymetrix 100K and 500K SNP chip arrays. It employs DM to make initial guesses and to form a prior for cluster characteristics. Clusters for each SNP are then re-calibrated in an *ad hoc *Bayesian manner; clusters populated with few data points, because of low minor allele frequency say, draw more influence from the prior. Because the laboratory effect resulted in too much across-study-variability, Affymetrix did not use HapMap as training. With their most recent product, the 6.0 array, Affymetrix provides yet another algorithm: Birdseed [23].

In the last year, Carvalho and colleagues [24] developed a pre-processing algorithm designed to remove the bulk of the lab effect. This treatment of the input permitted the use of HapMap as training data. As with BRLMM, an empirical Bayesian method is used to inform lowly populated clusters. The resulting algorithm is referred to as the Corrected Robust Linear Model with Maximum Likelihood Classification (CRLMM).

The goal in SNP calling algorithm design, thus far, has centred solely on increasing the number of SNPs that can be called with high confidence of accuracy. Little attention has been paid to developing measures of confidence. Each method provides its own metric with no standardization across algorithms. Worse, none of the metrics are explicitly linked to per-SNP accuracy. Questions as to whether metrics from the same algorithm translate to different accuracies based on the quality of the chip experiment remain open. Geneticists hoping to set an accuracy threshold to take SNPs forward for further genetic analysis are left in the dark.

The first goal of this paper is to describe and validate new features of our algorithm for SNP calling. Treatment of the input data with SNP Robust Multiarray Average (RMA) does not completely eliminate laboratory specific effects; we extend CRLMM to include a recalibration step using the original Bayesian framework to adjust clusters to account for these residual effects. We have also added a procedure that explicitly ties metrics of call confidence to per-SNP accuracy in a manner robust to chip-run quality. Details of both novel features can be found in the Materials and methods section. Because we are the developers of CRLMM, and have no plans of maintaining the original version, we avoid the use of yet another acronym and refer to our new algorithm as CRLMM as well. A software implementation of CRLMM is freely available through the oligo package at Bioconductor [25,26], an open development software project running under the statistical computing program R [27,28].

Our second goal is to describe validation benchmarks to be used in the future by other software developers for comparison purposes, as has been done for expression array algorithms with *affycomp *[29]. Beyond improving the genotype calling of existing chip arrays, this work lays down objectives and conventions to guide the development of future algorithms for calling emerging arrays of ever-increasing SNP density. The importance of sound assessment protocols is underscored by the recent formation of an NIH led effort to compare algorithms: the Genetic Association Information Network (GAIN) Alternative Calling Algorithms Working Group.

In the Results section we use our validation benchmarks to compare our new method (CRLMM) to the most widely used algorithm to date (BRLMM). We find that CRLMM provides more accurate genotype calls across datasets; at a high per-call accuracy, the drop rate of BRLMM is substantially higher than that of CRLMM across multiple datasets. Furthermore, CRLMM offers substantially improved estimates of accuracy. A less comprehensive comparison between CRLMM and Birdseed is included in the Discussion. Note that as more 6.0 data becomes publicly available, we will readily perform all our assessments on the new data and publish results on our SNPaffycomp website [30].

## Results

**Figure 1:**
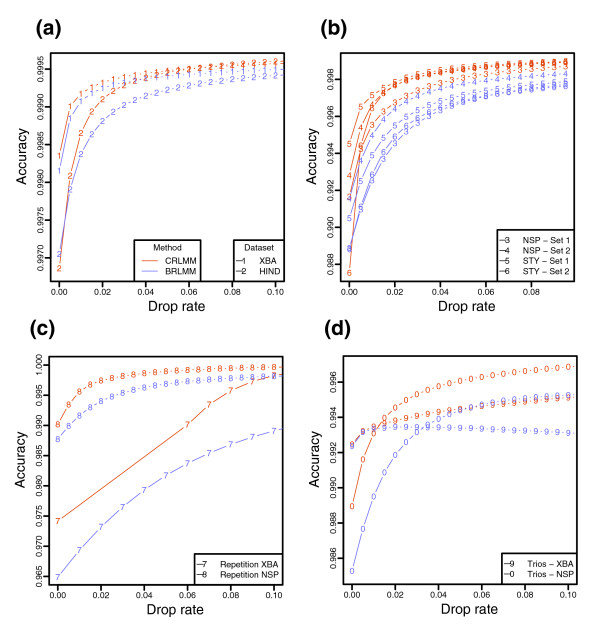
Accuracy versus ADPs for CRLMM (orange) and BRLMM (purple). Drop rates between 0 and 10% are examined. **(a) **ADPs are plotted for 269 HapMap samples hybridized by Affymetrix on 100K chips (XBA and HIND). Only high-quality hybridization data, as defined by Affymetrix, are used in this plot. The gold standard is HapMap calls. **(b) **ADPs are plotted for first-pass data from 152 and 95 samples hybridized on the 500K chips (Nsp and Sty) from the Broad Institute and Affymetrix, respectively. Again, the gold standard is HapMap calls. **(c) **ADPs are plotted for the 32 and 16 high quality replicates hybridized on XBA and Nsp chips. The consensus of the 32 and 16 high quality replicate chips is considered the gold standard for each chip type. Separate gold standards are derived from each calling algorithm result. These data were generated from the Chakravarti Lab. **(d) **ADPs are plotted for the 30 high quality trios hybridized on XBA and Nsp chips. Accuracy for trios is defined as percent of SNP trios that are Mendelian consistent. The trios may not have any dropped calls. The data were generated from the Chakravarti Lab.

The development of CRLMM involved training on the high quality Affymetrix HapMap array sets using HapMap Project genotypes as the correct calls [4]. CRLMM and BRLMM were then applied to high quality published Affymetrix HapMap data and newer first pass HapMap array data from the Broad Institute and Affymetrix. To compare the two algorithms, we generated accuracy versus drop rate plots (ADPs). Specifically, each point in the graph represents the proportion of calls above a given quality threshold in agreement with the HapMap Project. The quality threshold, which is based on the program-specific confidence metrics, is set such that the fraction of calls beneath it is the drop rate (of note, we use '1 - distance ratio' as the BRLMM confidence metric). CRLMM outperforms BRLMM in accuracy over a wide range of dropped call rates using the gold standard from the HapMap Project genotypes (Figure 1a,b). This result holds not only for the high quality Affymetrix set (Figure 1a; Figure S1 in Additional data file 1), which is expected since CRLMM was trained on it, but also for the first pass data (Figure 1b) and for data stratified into homozygote and heterozygote calls (Figure S1 in Additional data file 1).

One may critique the above result by pointing out CRLMM has an unfair advantage over BRLMM for the datasets used; CRLMM was trained on data generated from HapMap individuals. Moreover, the calls from the HapMap Project are known to have an error rate of their own. To examine whether CRLMM outperforms BRLMM for array data from other individuals, the two algorithms were applied to a set including both multiple replicate and trio samples on the Xba and Nsp chips of the 100K and 500K arrays, respectively. The replicate and trio files were run together because running the former files exclusively would be highly artificial, making results ungeneralizable. The gold standards for the replicate data were the majority call among high quality chips. Accuracy was then defined as agreement with these consensus sets, which were generated separately for each dataset and calling method. The trio data were scored by tabulating the fraction of SNP trios (mother, father, and child) not violating Mendelian inheritance among those without any dropped calls. As with the HapMap validation, the accuracy for these two types of files was examined at different drop rates. The ADPs of both the replicates and trios demonstrate that CRLMM makes calls more accurately than BRLMM on non-HapMap sets (Figure 1c,d). These data afford alternative ways of comparing algorithms since large datasets with independent verification by multiple genotyping modalities do not exist other than those relating to the HapMap Project.

An alternative, quantitative presentation of the same data above can be found in Table 1. For the Affymetrix first-pass Sty set, we eliminated all SNPs with a predicted per-call accuracy less than 0.995 and calculated the average accuracy of the remaining data to be 0.99915. Applying this average accuracy to other datasets as a rough approximation to a per-call accuracy of 0.995, CRLMM achieves a drop rate of 0.6 to 24% in the datasets examined. The datasets incurring high drop rates for CRLMM include poor quality chips; these same sets yield drop rates of greater than 50% for BRLMM. Setting aside these datasets, CRLMM's drop rate was 2 to 7 times lower than for BRLMM (4.46 to 2.28% and 42 to 5.65%, respectively).

**Table 1:** Drop rate at an average accuracy of 0.99915

	Drop rate		
		
Dataset	BRLMM	CRLMM	Relative rate
Affymetrix high-quality XBA arrays	0.0115	0.00575	2.00
Affymetrix high-quality HIND arrays	0.0446	0.0228	1.95
Affymetrix high-quality STY arrays	0.0873	0.0171	5.10
Broad Institute first-pass STY arrays	-	0.241	-
Affymetrix first-pass STY arrays	-	0.110	-
Affymetrix high-quality NSP arrays	0.086	0.0347	2.48
Broad Institute first-pass NSP arrays	-	0.180	-
Affymetrix first-pass NSP arrays	-	0.231	-
Chakravarti XBA replicate arrays	-	0.110	-
Chakravarti NSP replicate arrays	0.42	0.0565	7.43

Drop rates greater than 0.50 to reach the desired average accuracy are not considered.

**Figure 2:**
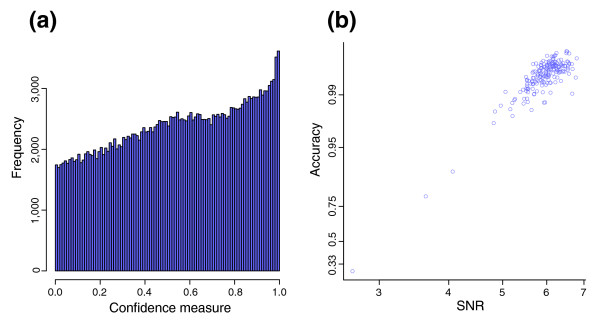
Accuracy prediction plots for Affymetrix first pass Sty HapMap samples. **(a) **A histogram of the BRLMM confidence measure is plotted for a sample chip with an average accuracy lower than 33% called by either BRLMM or CRLMM. **(b) **The graph shows a scatter plot of average accuracy of chips as called by BRLMM versus SNR. The y-axis is in the logit scale; the x-axis, the log scale.

CRLMM allows for the identification of these poor quality chips. It is well known that the inclusion of poor quality chips in a dataset may distort calling algorithms to such a degree that mistaken calls are made even on high quality chips. Therefore the identification and exclusion of poor quality chips is vital in any analysis. In this regard, BRLMM proves to be inadequate; using a summary statistic based on BRLMM confidence metrics will not accurately reflect the chip quality. As an example, consider one of the samples in the Affymetrix first pass Sty data; measured against the HapMap as the gold standard, it has an average accuracy less than 33% whether it is called by BRLMM or CRLMM. This degree of accuracy can be achieved by guessing, which implies that no information is provided by the array. Yet, Figure 2a demonstrates that BRLMM calls 10,000 SNPs at a very high confidence level (confidence measure >0.95). The implication is that the BRLMM confidence measure cannot be used to gauge the overall quality of a chip, because its meaning is distorted for poor quality chips; in fact, Affymetrix suggests the use of DM to exclude poor quality chips before applying BRLMM. On the other hand, the signal to noise ratio (SNR) measure we have developed (see Materials and methods) is an excellent predictor of chip-specific accuracy (Figure 2b).

**Figure 3:**
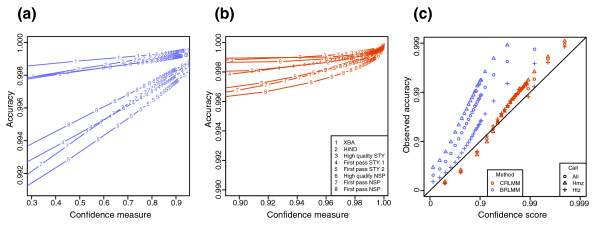
Robustness to bad quality chips. Accuracy is plotted against confidence thresholds for various datasets. In other words, the data in Figure 1 are plotted again except that the confidence measures used previously to achieve specified drop rates are now placed on the x-axis. Results of all HapMap datasets are shown from **(a) **BRLMM and **(b) **CRLMM. **(c) **Accuracy versus confidence plots (ACPs) are made for BRLMM (purple) and CRLMM (orange). The points are further stratified by call type according to the HapMap gold standard. The STY and NSP are the array types described in the text. Hmz and Htz are abbreviations for homozygous and heterozygous, respectively.

Not only is the BRLMM confidence metric invalid for poor quality chips, it corresponds to different accuracies from dataset to dataset. Figure 3a,b show plots similar to the ADPs as before for datasets of different quality and from different labs; however, the drop rate is replaced with confidence metric thresholds on the abscissa. The plot for BRLMM (Figure 3a) shows a wide variation in accuracy across different datasets for any given confidence threshold. The implication of this finding is that a BRLMM confidence threshold found to give an acceptable accuracy rate in distal analyses for one dataset may not apply to another set. In contrast, the plot for CRLMM (Figure 3b) demonstrates that its confidence measure has greater robustness to laboratory and chip quality effects.

Ultimately, the end-user must have a per-call measure of accuracy to identify which SNPs to exclude from further analysis. Labs using BRLMM are left to their own devices to connect the program's confidence metric to accuracy. In Figure 3c, we divide the data by quantiles with respect to the confidence metric and make average accuracy versus average confidence plots (ACPs). The ACPs for BRLMM derived data show that connecting the confidence metric to accuracy is not straightforward, because the metric appears to correspond to different accuracies depending on whether the call is heterozygous or homozygous. The ACPs for CRLMM on the other hand do not show this difference. In fact, that the plot closely follows the diagonal demonstrates the CRLMM confidence metric may be treated as the predicted per-call accuracy. ACPs comparing BRLMM and CRLMM results for other datasets are shown in Figure S2 in Additional data file 1.

## Discussion

The first step in a useful genotyping algorithm is transforming raw data into genotype calls. As we have demonstrated, the quality of these calls can vary. A second step in a useful genotyping algorithm is to quantify how certain we are about our calls. We have improved our previous algorithm [24] as a solution to step one. A naive approach for step 2 was described in that work as well, but our comparison tool demonstrated that it did not perform well (data not shown). In this paper we additionally develop a more sophisticated version of the second step and demonstrate important practical implications.

**Figure 4:**
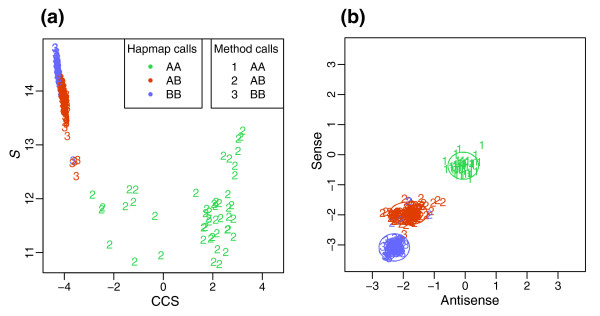
Genotype regions. These plots display the space on which clusters are assigned genotypes. Colors represent HapMap gold standard calls; numbers represent the calls made by the array algorithms. For SNP SNP_A-1676170, **(a) **BRLMM genotype regions are shown in the Contrast Center Stretch (CCS) space. The x-axis is a contrast measure that captures the relative intensity difference of allele A with B. The y-axis is related to the average intensity from the A and B alleles. See the BRLMM white paper for more details [22]. For the same SNP, **(b) **CRLMM regions are plotted. The log ratios of allele A to allele B from intensities derived from probesets of the sense (y-axis) and antisense (x-axis) orientation are shown (that is, *M*_+ _versus *M*_- _plot). The ellipses represent the cluster regions obtained from the HapMap training set.

Across datasets from multiple laboratories and by different methods of validation, CRLMM is just as, and in many cases more, accurate than BRLMM in calling genotypes from Affymetrix SNP chips. This result is due in large part to the utilization of HapMap information in making calls. Figure 4 shows a SNP for which the intensities in BRLMM's Contrast Center Stretch space [22] clusters poorly in comparison to the CRLMM cluster regions formed from training on HapMap data. The HapMap influence is built into the CRLMM algorithm; calls are informed by the high-quality HapMap data without users having to seed their input data with files generated from the project. In addition, the greater accuracy at higher dropped call rates, as observed in Figure 1a, is due to the greater discriminating power of the CRLMM confidence metric to predict which calls are more likely to be correct.

An apparent improvement of BRLMM over DM is that the former no longer has a propensity to drop heterozygous calls at a given confidence threshold. To assure equal drop rates for homozygous and heterozygous calls, we found that Affymetrix altered the confidence metric. The end result is that the metric corresponds to different accuracies for homozygous as compared to heterozygous calls (Figure 3c). We also believe this feature results in artefact that explain the slight reduction in accuracy with increased drop-rates seen for BRLMM in Figure 1d: for higher accuracy calls, errors are more confounded with call types (heterozygous or homozygous), resulting in more Mendelian inconsistencies. We do not implement such an approach because the greater difficulty in calling a heterozygous genotype is an intrinsic characteristic of the array technology. Our approach is to report an accurate confidence measure rather than one that assures equal drop rates.

It is true that the use of a single cut-off for CRLMM derived data will incur more heterozygous drop-out (Figure S3 from Additional data file 1), a result that may bias distal analyses. Nevertheless, uniform drop rates can still be easily achieved by choosing a more stringent confidence cut-off for homozygotes; in this way, calls can be both above a pre-specified accuracy threshold and have equivalent drop rates between homozygotes and heterozygotes. One may wonder whether these steps can be legitimately applied to results from an algorithm, since calls and confidence metrics are not made with absolute certainty. Figure S4 from Additional data file 1 demonstrates this concern to be largely unfounded. Figure S5 from Additional data file 1 shows that even after forcing drop rates to be the same between heterozygotes and homozygotes, CRLMM results at different accuracy thresholds are still more accurate than BRLMM. In the end, we do not view the equalization of drop rates to be a definitive solution to this problem; rather, modifications to statistical methodologies for association and linkage studies are required to account for this factor.

Just as important as accuracy are assessments of quality for both chips and calls. We have shown that the BRLMM confidence metric is inadequate for chip quality determination, corresponds to different accuracies from dataset to dataset, and even has different meanings for homozygotes and heterozygotes on the same chip. Regarding the assessment of chip quality, it should be noted that Affymetrix recommends running DM to eliminate poor quality chips prior to using BRLMM [22]. For CRLMM, this step is trivial; chips with SNRs below 4.5 and 2.36 for the 100K and 500K chips, respectively, should be excluded from further analysis.

In theory, a confidence metric may be defined over any space. One may believe that so long as it proves to be stable over datasets and call types, it will be of optimal utility. All an end-user needs to do is consult an ADP plot to set an accuracy threshold over which SNPs can be taken forward for further analysis. This notion is false. The reason is that the accuracy so calculated is an average of individual SNPs with corresponding variable accuracy. Within this range, there will be a significant portion of individual SNPs of lower accuracy than the average value. To address this problem, we made CRLMM's confidence metric per-call accuracy. We feel methodologies developed in the future will be of greater utility if this convention is followed.

**Figure 5:**
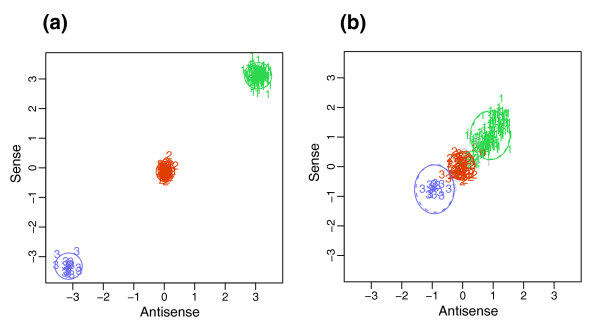
Comparison to Birdseed. **(a) **As Figure 1 but for 6.0 data and Birdseed instead of BRLMM. **(b) **As Figure 3 but for 6.0 data and Birdseed instead of BRLMM.

It is not inconceivable that new methods of genotype calling that surpass CRLMM will be devised. Moreover, Affymetrix will continue to design new SNP arrays; adaptations of old algorithms for these chips will require additional validation. Algorithms of the future may prove to be more accurate, as demonstrated by ADPs, and have confidence metrics even more reflective of true accuracy, as exhibited by ACPs. In fact, the most important aspect of this work is laying the ground work for a standard set of assessments by which different methodologies may be measured against each other. Indeed, we already have included comparisons of CRLMM to Birdseed (Affymetrix default algorithm for 6.0 array). Figure 5 shows two of our assessments performed on HapMap samples analyzed with the 6.0 array. CRLMM continues to perform better and, in particular, demonstrates better performance across laboratories.

The datasets used in this study is freely available at our SNPaffycomp website [31] at which one will also be able to review results of different algorithms. The establishment of standard benchmarks for Affymetrix SNP chip analysis is analogous to that already done for the sister technology of Affymetrix expression arrays, the so-called Affycomp [29].

## Materials and methods

### Datasets

We used several HapMap datasets representing runs of varying quality, different labs, and different times. The publicly available high quality HapMap Project data are on 269 individuals genotyped on 100K arrays [32] by Affymetrix [4]. High quality 500K [33] and 6.0 chip data run by Affymetrix are also available on all 270 HapMap samples [30].

Another two sets of 500K chip results from 95 and 152 HapMap individuals were provided to us by Affymetrix and the Broad Institute, respectively; these data differed from the aforementioned sets principally in that they were derived from first pass runs of variable albeit typical quality. Two 6.0 array datasets, from 44 and 96 HapMap samples, were run at the Chakravarti laboratory for testing purposes.

The other Chakravarti laboratrory datasets were generated from non-HapMap individuals. A replicate dataset composed of 40 50K Xba chips (part of the 100K chipset) run on a single DNA sample were used as well as 30 trios. Eight of the 40 replicates were of low quality as assessed by SNR. Similarly for the 250K Nsp chip (part of the 500K chipset), the Chakravarti lab provided a replicate dataset of 16 chips and 30 trios. All 16 replicates were of high quality. All trio data for both the Xba and Nsp chips were of high quality. Replicates were performed with the same DNA prep but all subsequent steps - ligation, amplification, fragmentation and hybridization - were repeated on different chips.

### Measures of accuracy

The HapMap sample gold standard against which outputs from either BRLMM or CRLMM are compared is derived from the final HapMap Project genotype calls (data release 22) made on a number of platforms other than Affymetrix arrays [4]. Only a small fraction of SNPs represented on Affymetrix arrays are not typed in the HapMap Project. Though highly accurate in total, HapMap Project calls are by no means perfect. Nevertheless, there is no expectation that mistakes in HapMap should favor one genotype calling method over another. There are several SNPs for which the allele names are obviously reversed between HapMap and Affymetrix and, thus, these SNPs were corrected by hand.

For the replicate data, high quality chips (operationally defined as having an SNR greater than 4.5 and 2.36 for the 100K and 500K chips, respectively, as explained above) are used in deriving the gold standard. For each SNP, calls for the three genotypes are tallied across the replicates. The genotype comprising greater than 50% of the calls is designated the gold standard call. SNPs not meeting this criterion are excluded from validation. Distinct gold standards are generated for each chip and method.

In both the HapMap and replicate data validation, accuracy versus dropped rate curves are plotted. Each point on the plots represents the mean number of SNPs correctly called ignoring a specified percent of the lowest quality SNPs. Quality is assessed by program specific metrics of confidence, that is, 1 - ratio distance and percent accuracy for BRLMM and CRLMM, respectively.

For trio data, family structures are exploited to measure the accuracy of genotype calling. The number of Mendelian errors is tabulated at a given dropped call rate. The resulting value is subtracted from and divided by the number of SNP trios that have no dropped calls to give an accuracy rate.

### Pre-processing and genotyping algorithm

A brief summary of the pre-processing and genotyping algorithms is presented here; for a more technical treatment, see Carvalho *et al*. [24]. Starting with the feature level data available in the CEL files provided by Affymetrix, we summarize the probes associated with each SNP in a manner similar to RMA [34]. The resultant values are proportional to the log_2 _of the quantity of DNA in the target sample associated with alleles *A *and *B*. Sense and antisense information are kept separately to allow the correct calling of the genotype by one strand when the other is non-informative [24]. We denote these values as θ_A,-_, θ_B,-_, θ_A,+_, θ_B,+_, and transform them into the log ratio *M*_- _= θ_A,- _- θ_B,- _and *M*_+ _= θ_A,+ _- θ_B,+ _and average log intensities *S*_- _= (θ_A,- _+ θ_B,-_)/2 and *S*_+ _= (θ_A,+ _+ θ_B,+_)/2.

**Figure 6:**
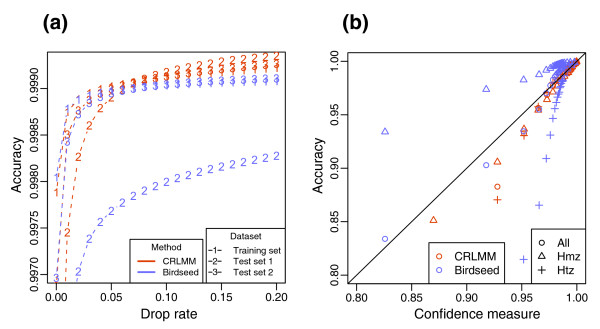
Comparison of SNP quality. CRLMM regions are plotted in log ratio space for **(a) **a high quality SNP (SNP_A-1750453) and **(b) **a low quality SNP (SNP_A-1709733). Hmz and Htz are abbreviations for homozygous and heterozygous, respectively.

**Figure 7:**
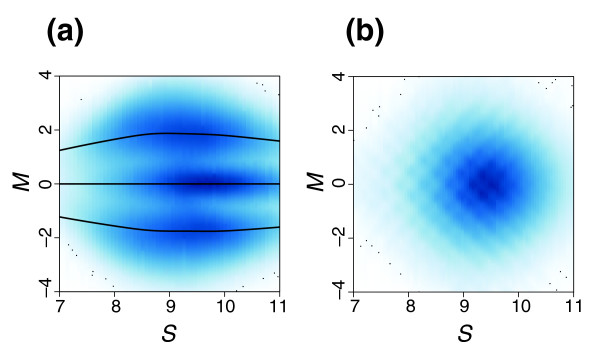
Log intensity ratios (allele A versus B), denoted with *M*, for all SNPs on one chip plotted against average log intensity *S *values. Both sense and antisense values are shown in all plots. A scatter-plot of these data would include 500,000 points and thus would be hard to interpret. We therefore show two-dimensional histograms with dark and light shades of blue indicating the existence of many and few points, respectively. **(a) **High quality array. **(b) **Low quality array.

We denote the log-ratio of SNP *i *from sample *j *by *M*_*i*,*j *_with sense and antisense orientation denoted by *s *= +,-. We code the genotypes by *k *= 1, 2, 3 for AA, AB, and BB, respectively. In general, *M *values impart strong discriminatory power (Figure 4), though there is SNP to SNP variation (Figure 6). Also, SNPs with inferior separability are associated with long target fragment lengths or extreme values of *S*, which is demonstrated in Figure 7a. We describe these effects with a simple mixture model. To simplify the fitting procedure we estimate the model separately on each array and treat the sense and antisense features as exchangeable. We therefore drop the *j *and *s *notation and write:

[*M*_*j *_| *Z*_*i *_= *k*] = *f*_*k*_(*X*_*i*_) + *e*_*i*,*k*_

where the *X*_*i *_represents covariates known to cause bias, *f*_*k *_describes the effect associated with these covariates, and *e*_*i*,*k *_captures the error term, which we assume to be a random normal variable with mean 0 and constant variance. Further, we assume *f*_*1*_ = *f*_*3*_ and *f*_*2*_ = *0*.

Motivated by Figure 7a, we include fragment length *L *and the average intensity *S *as covariates and model:

*f*_1_(*L*_*i*_;*S*_*i*_) = *f*_*L*_(*L*_*i*_) + *f*_*S*_(*S*_*i*_)

with *f*_*L*,_ as a cubic spline having three degrees of freedom and *f*_*S*_ as a cubic spline having five degrees of freedom. We fit the model using the Expectation-Maximization (EM) algorithm. Examples of the estimated *f*_*L*_ and *f*_*S*_ are included in Figure 7a. A high quality hybridization will separate genotypes, that is, the signal *f*_*1*_ will be larger than the standard deviation of errors *e*.

The fitted models can also be used to obtain genotype calls by estimating and maximizing the probability of each class for each SNP. However, for all SNPs for which we have HapMap calls (available for about 96% of SNPs on the arrays), we use a supervised learning approach, which yields more accurate genotype calls. We use these calls to define 'known' genotypes, which in turn permits us to define a training set. For SNPs with no HapMap calls we use the estimates from the model described above to define the 'known' classes. With the training data in place we use a two-stage hierarchical model and give likelihood-based closed-form definitions of the genotype regions. For each SNP, we define two-dimensional genotype regions based on the sense and antisense *M *values. The utility of the hierarchical model is most apparent for SNP regions for which there are few observations in the training step. Using empirically derived priors for the centers and scales of the genotype regions, we give a closed form empirical Bayes solution to predict centers and scales for cases with few or no observations.

Let *Z*_*i*,*j *_be the unknown genotype for SNP *i *on sample *j*. Figures 4b and 6 demonstrate that the locations of these genotype regions are SNP specific. Furthermore, these pictures suggest that the behavior of the log-ratio pairs can be modeled by bivariate normal distributions. We use a two-level hierarchical multi-chip model with the first level describing the variation seen in the location of genotype regions across SNPs and the second, the variation seen across samples within each SNP. The model can be written out as:

[*M*_*i*,*j*,*s *_| *Z*_*i*,*j *_= *k*;*m*_*i*,*k*,*s*_] = *f*_*j*,*k*_(*X*_*i*,*j*,*s*_) + *m*_*i*,*k*,*s *_+ *e*_*i*,*j*,*k*,*s*_

Here, *X*_*i*,*j*,*s *_and *f*_*j*,*k *_are as above but with the *j *and *s *notation re-introduced, *m*_*i*,*k*,*s *_is the SNP-specific shift from the typical genotype region centers, and *e*_*i*,*j*,*k*,*s *_represents measurement error. We expect different samples to have different biases; thus, the effects function *f *now depends on *j*. Notice that the SNP-specific covariates *X *also depend on the sample because the average signal *S *may vary from sample to sample. The '*m*'s represent the cluster center shifts not accounted for by the covariates included in *X*. To define the first level of our model, we denote the vector of SNP-specific region centers with m_*i*_* = *(*m*_*i*,*1*,+_, *m*_*i*,*2*,+_, *m*_*i*,*3*,+_, *m*_*i*,*1*,-_, *m*_*i*,*2*,-_, *m*_*i*,*3*,-_). We model the distribution of this vector with a multivariate normal distribution. Notice that, by definition, m is centered at 0, since the mean levels of the three genotypes are absorbed into *f*. The second level of the model, the variability seen within the genotypes for each SNP, is described by the '*e*'s. We assume these to be independent (conditioned on genotype *Z*) normally distributed random variables across samples and SNPs. We use an inverse chi-squared prior to improve estimates of the variance structure when not enough data are available. Because the large number of SNPs permit us to estimate the *f*_*j*_s precisely, for simplicity, we treat them as known. With this estimate of *f*_*j *_in place for each sample, all we need to make our likelihood-based genotype calls are estimates of the centers and scales. The key idea is to consider the HapMap calls as known genotypes and use this information to obtain maximum likelihood estimates. A second step is to update these estimates with posterior means derived from the hierarchical model. The mathematical details are described in Carvalho *et al*. [24].

The next step is to make a genotype call and calculate a confidence measure for any given pair (sense and antisense) of observed log-ratios: *M*_*i*,*j*,+ _and *M*_*i*,*j*,-. _Notice that these *M *values can come from any study, and we will use the centers and scales, estimated from the HapMap data. We do this by forming a likelihood based distance function based on the mixture model described above. Our prediction is the genotype *k *that minimizes the negative log-likelihood. Furthermore, the log likelihood ratio test serves as a predictor of confidence accuracy.

Although our pre-processing procedure greatly improves comparability across lab/studies, some slight differences in cluster centers appear to persist. For this reason we add an extra step to the algorithm described in Carvalho *et al*. [24]. After obtaining genotype calls, we use those achieving log-likelihood ratios associated with 99% concordance rates and repeat the previously described steps to recalculate the centers and scales. We then compute calls and log-likelihood ratios using these new parameters.

### Measure of chip quality

As a measure of chip quality we define a SNR that captures the difference of the intensity values among the genotype clusters across all SNPs of a given chip. The fitted models described in the previous section are used to define our metric:

SNR = median_i_{ f_1_(X_i_) }^2^/{ average_k_ [ var(*e*_*i*,*k*_) ] }

Figure 7a,b show data for high and low quality arrays, respectively. In the low quality array (Figure 7b), the fact that the variability of the errors *e *overshadow the signal *f*_*1*_ can be appreciated by noticing the loss of three distinct horizontal bands, corresponding to the three genotypes as seen in Figure 7a. In the low quality array, information about genotypes is lost. Figure 2b shows that this value correlates well with sample specific accuracy. Moreover, chips with substantial fractions of dropped calls by DM (>10%) yield lower SNRs (data not shown). By empirical methods, we arrived at a high-quality chip SNR threshold of 4.5 for 100K chips and 2.36 for 500K chips.

### Confidence measures

The measures of confidence of existing calling algorithms are, by and large, all based on a similar approach. Specifically, once genotype regions are defined for a given SNP, the measure of confidence for a given call is derived from the distance between: the point related to the call; and the region centers. This is the basis of the distance ratio used in BRLMM [22] as well as the likelihood based function described by [24]. However, this measure ignores other important predictors of accuracy such as chip quality, SNP quality, and the genotype.

For chip quality, we use the aforementioned metric SNR. To capture SNP quality, we quantify the distance between the regions for the three genotypes. A variety of factors can affect the efficacy of the feature probes in determining the genotype for a given SNP. Clearly, SNPs with greater separation of regions can be called more accurately (Figure 6a,b). We capture this notion by calculating the following value for each SNP: minimum distance between the center of region AA and AB and the center of AB and BB.

Finally, we noted that accuracy is slightly different for homozygous and heterozygous genotypes. Heterozygous genotypes appear to be more difficult to call because their corresponding regions are more liable to be abutting the two homozygous regions. Therefore, a greater proportion of heterozygous points are close to the periphery of homozygous regions, where they are incorrectly called.

To provide a useful prediction of accuracy that takes all these measures into account, we perform a logistic regression:

*Logit*{ *Pr*(Correct Call | Covariates) } = *f*_*1*_(Distance, SNP Quality) + *f*_*2*_(SNR)

We assume the *fs *are smooth and model them with cubic splines. We fit this model on the high quality HapMap dataset to obtain estimates of *f*_*1*_ and *f*_*2*_. The model was fit to heterozygous and homozygous calls separately. The resultant covariates are used in new datasets to calculate per-SNP accuracy.

### Validation of the confidence metric

To ascertain the robustness of the confidence metric from datasets generated from different labs and of different quality, we plot accuracy versus confidence threshold. The thresholds correspond to values used to achieve the specified dropped rates in the ADPs. Confidence metrics that are more robust will produce plots with less outward fanning.

Another method of validating confidence metrics is to divide calls among quantiles according to the metric and plot average accuracy against the confidence metric for each quantile (the so-called ACP). Ideally, curves stratified by homozygote and heterzygote calls should not deviate from one another. Confidence metrics that accurately predicted per-call accuracy produce curves that lie on the diagonal.

## Abbreviations

ABACUS, Adaptive Background genotype Calling Scheme; ACP, average accuracy versus quantile confidence metric plot; ADP, accuracy versus drop rate plot; BRLMM, Bayesian Robust Linear Model with Mahalanobis Distance Classifier; CRLMM, Corrected Robust Linear Model with Mahalanobis Distance Classifier; DM, Dynamic Model; MPAM, Modified Partitioning Around Medoids; RLMM, Robust Linear Model with Mahalanobis Distance Classifier; RMA, Robust Multiarray Average; SNP, single nucleotide polymorphism; SNR, signal to noise ratio.

## Authors' contributions

SL and RAI conceived the study, developed the software, and drafted the manuscript. AC conceived the study, provided the data, and revised the manuscript. BC conceived the study, developed the software, and revised the manuscript. DEA conceived the design and revised the manuscript. DJC revised the manuscript.

## Additional data files

The following additional data are available. Additional data file 1 contains the supplemental figures.

## Supplementary Material

Additional data file 1 Supplemental figures Click here for file
