# Supplementary material for: Validation and extension of an empirical Bayes method for SNP calling on Affymetrix microarrays
Source: Genome Biol. 2008 Apr 3;9(4):R63. doi: 10.1186/gb-2008-9-4-r63 (PMC2643934; doi:10.1186/gb-2008-9-4-r63)
Supplement: Additional data file 1 — Supplemental figures [file gb-2008-9-4-r63-S1.pdf]

Supplemental Figure 1A

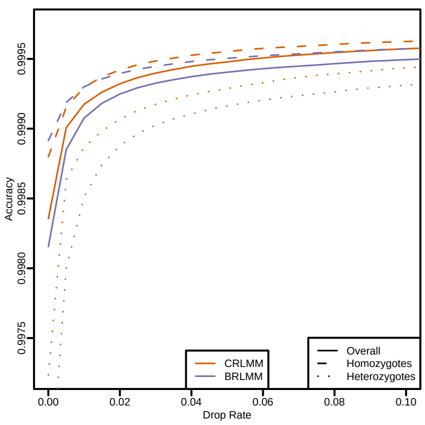

Supplemental Figure 1B

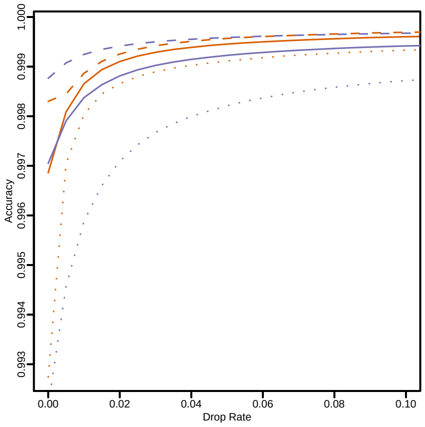

Supplemental Figure 1C

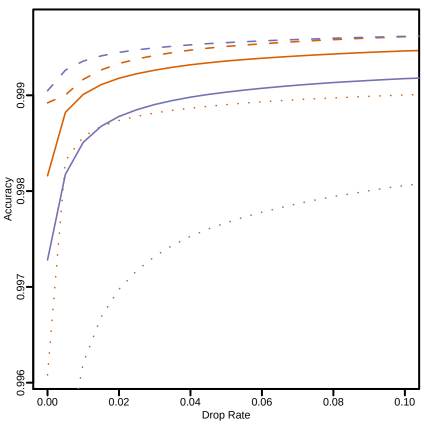

Supplemental Figure 1D

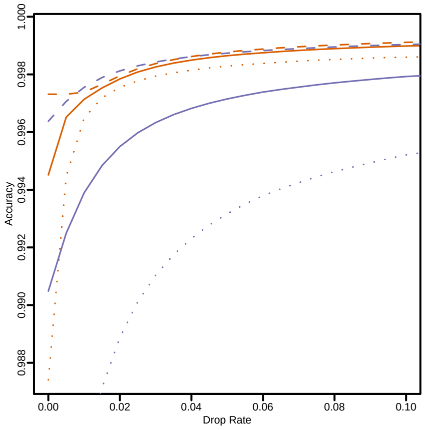

Supplemental Figure 1E

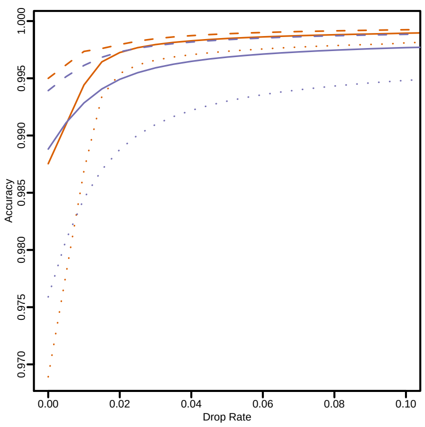

Supplemental Figure 1F

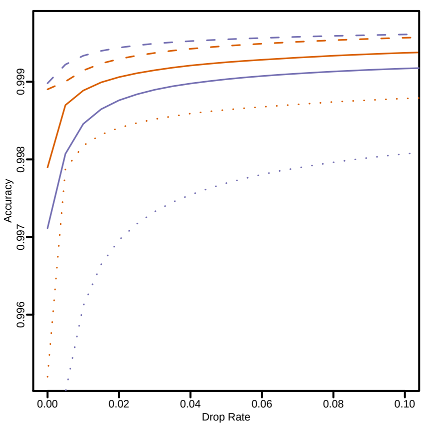

Supplemental Figure 1G

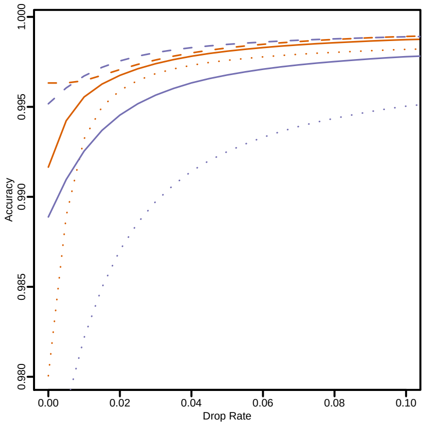

Supplemental Figure 1H

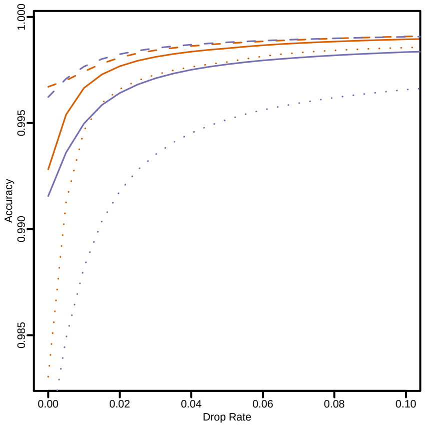

Supplemental Figure 2A

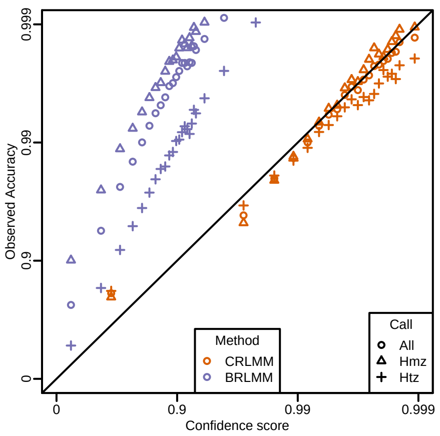

Supplemental Figure 2B

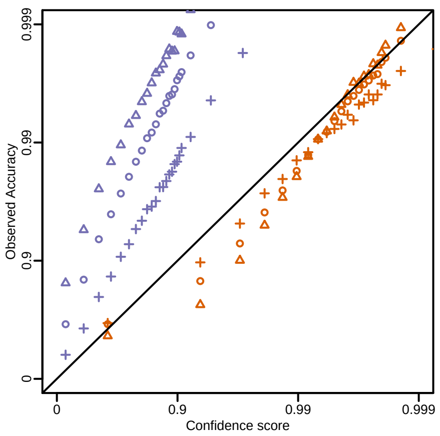

Supplemental Figure 2C

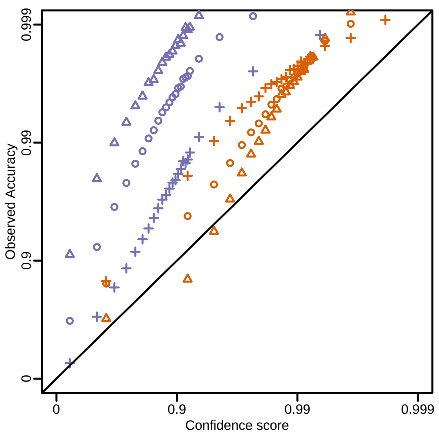

Supplemental Figure 2D

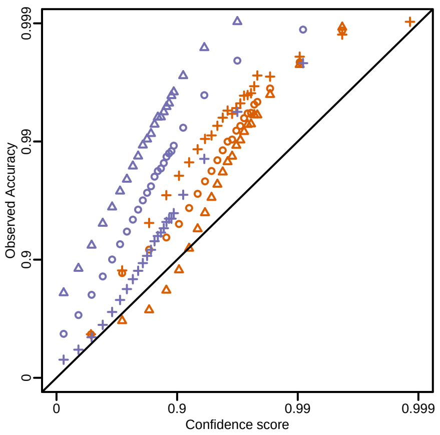

Supplemental Figure 2E

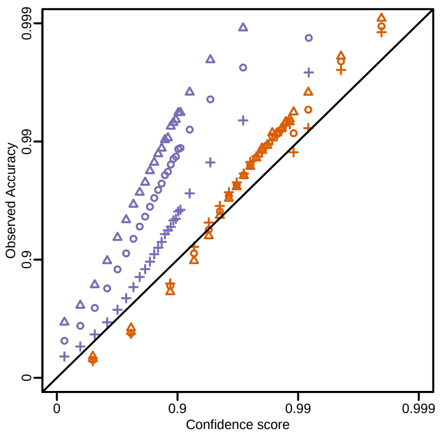

Supplemental Figure 2F

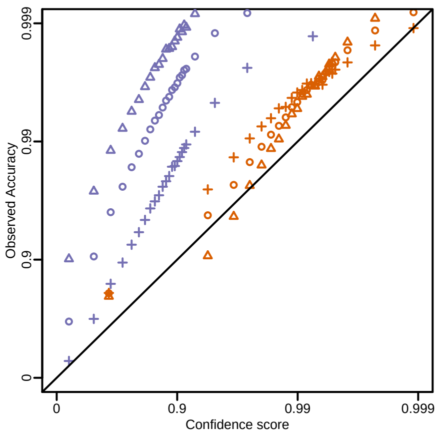

Supplemental Figure 2G

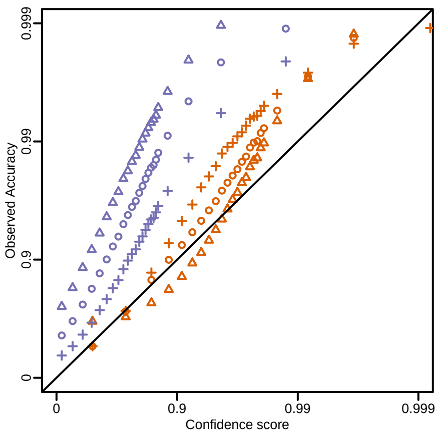

Supplemental Figure 2H

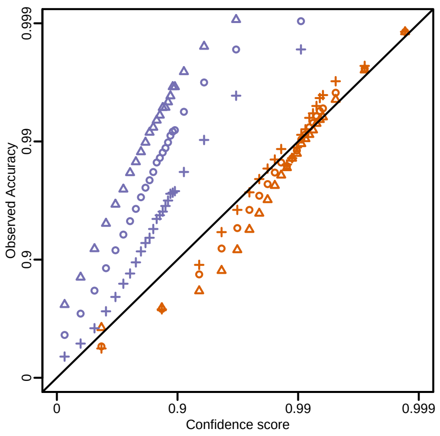

Supplemental Figure 3A

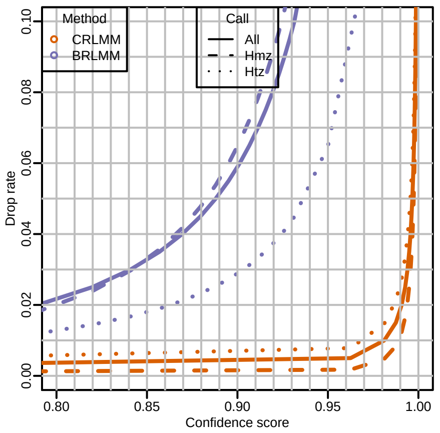

Supplemental Figure 3B

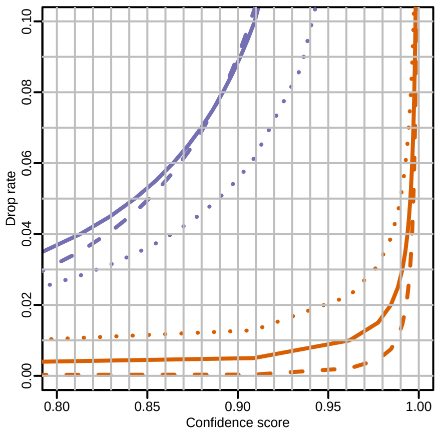

Supplemental Figure 3C

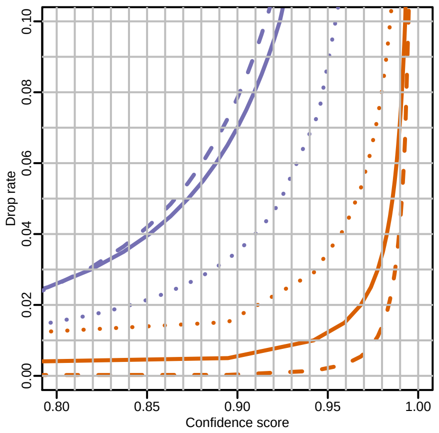

Supplemental Figure 3D

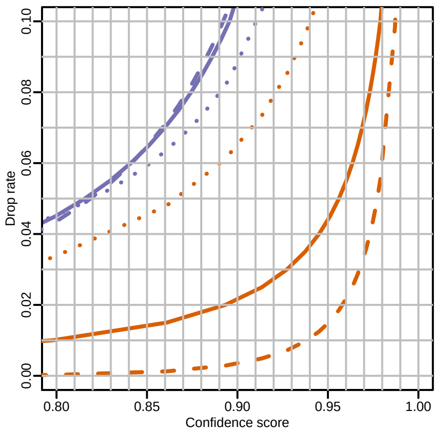

Supplemental Figure 3E

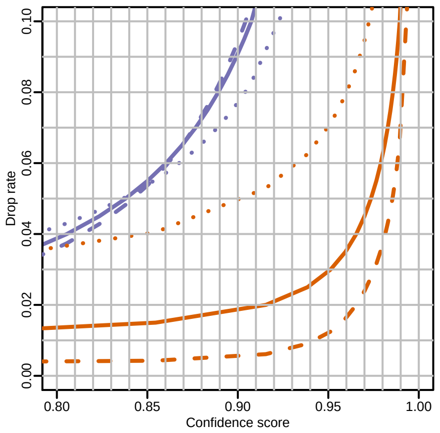

Supplemental Figure 3F

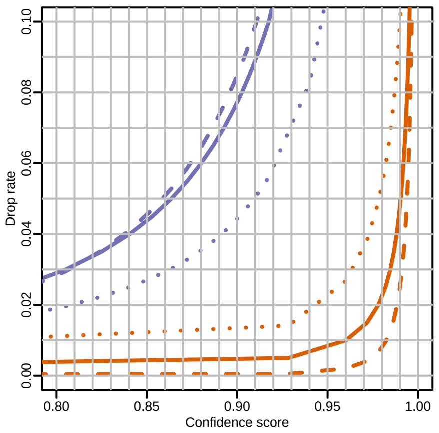

Supplemental Figure 3G

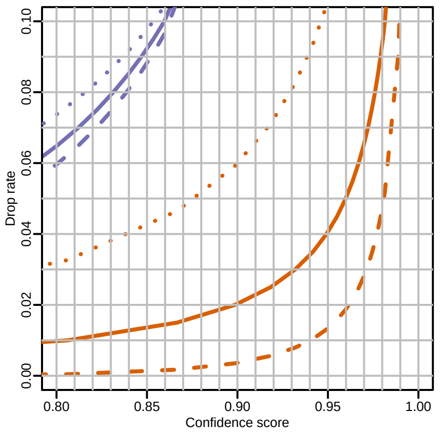

Supplemental Figure 3H

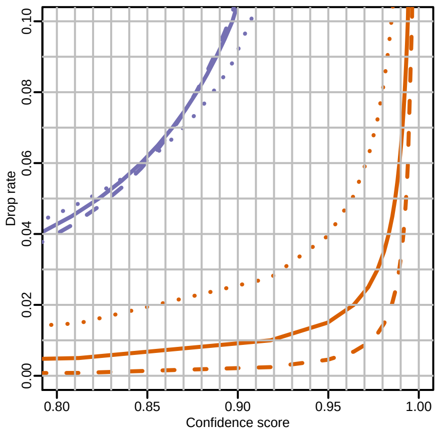

Supplemental Figure 4A

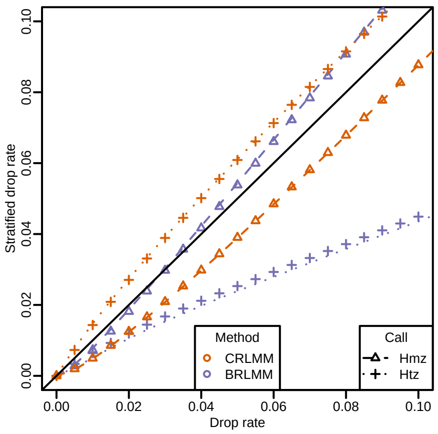

Supplemental Figure 4B

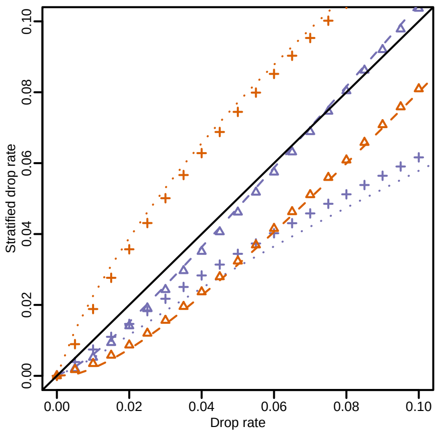

Supplemental Figure 4C

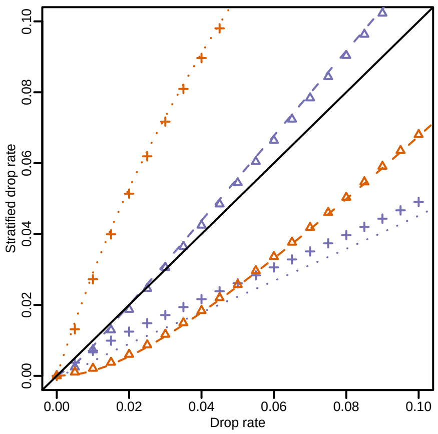

Supplemental Figure 4D

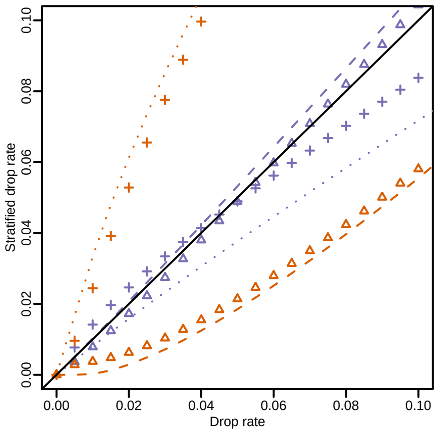

Supplemental Figure 4E

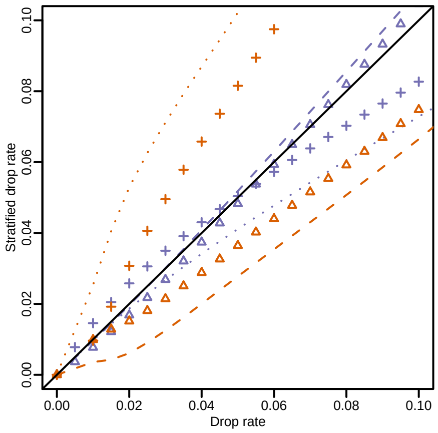

Supplemental Figure 4F

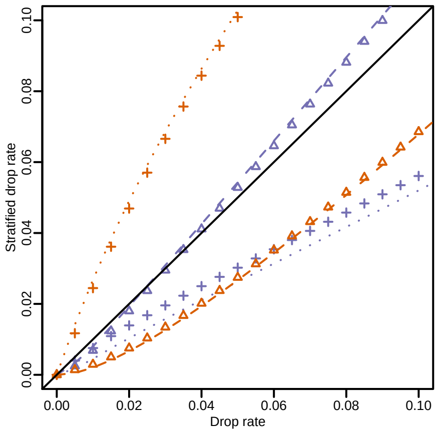

Supplemental Figure 4G

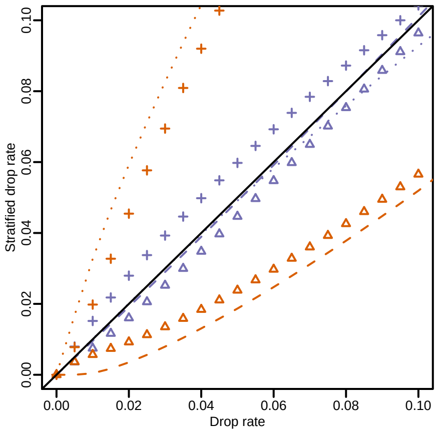

Supplemental Figure 4H

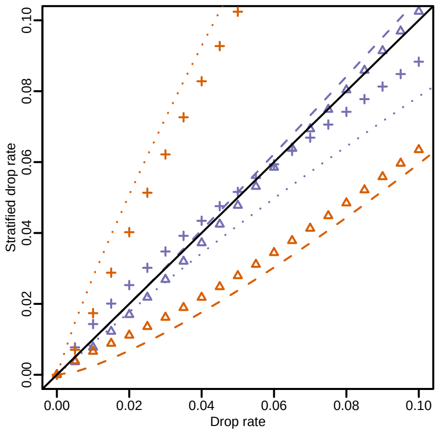

Supplemental Figure 5A

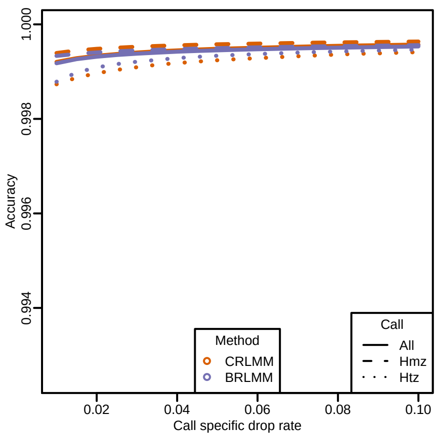

Supplemental Figure 5B

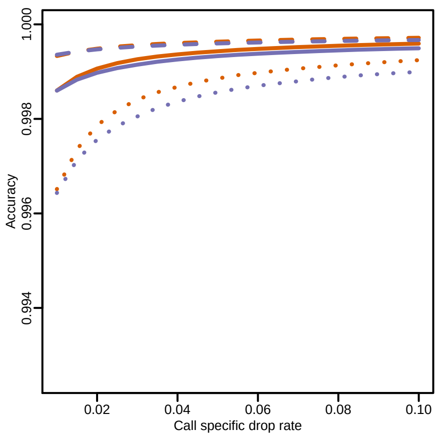

Supplemental Figure 5C

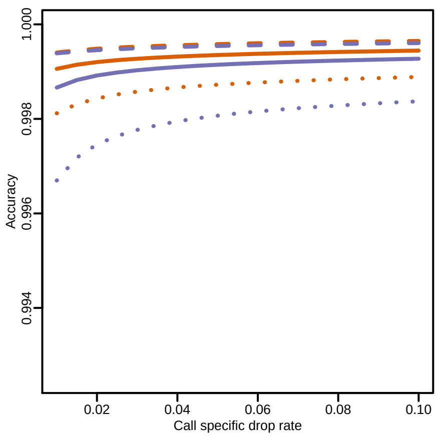

Supplemental Figure 5D

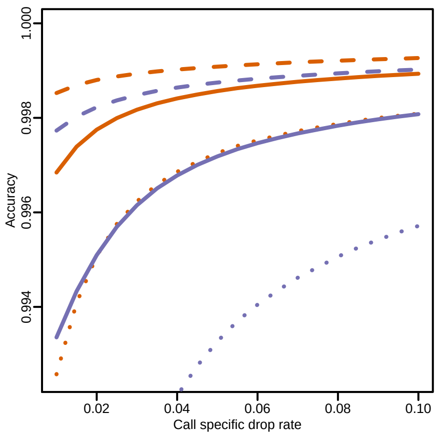

Supplemental Figure 5E

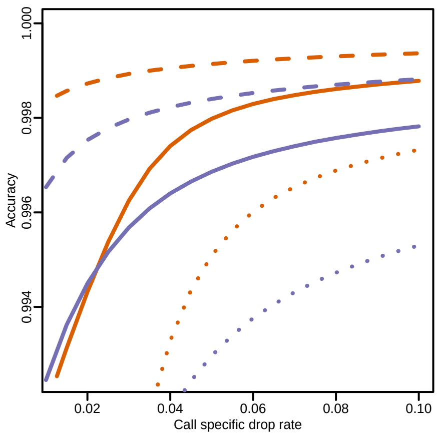

Supplemental Figure 5F

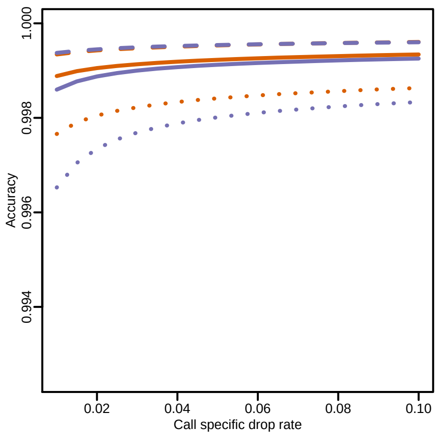

Supplemental Figure 5G

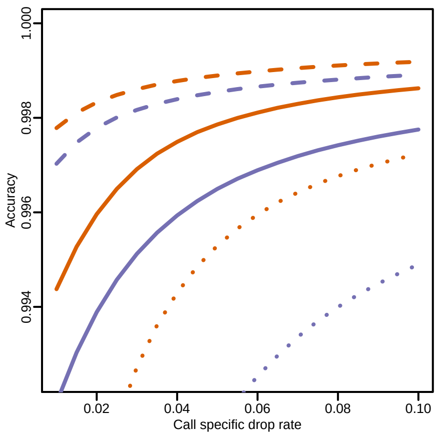

Supplemental Figure 5H

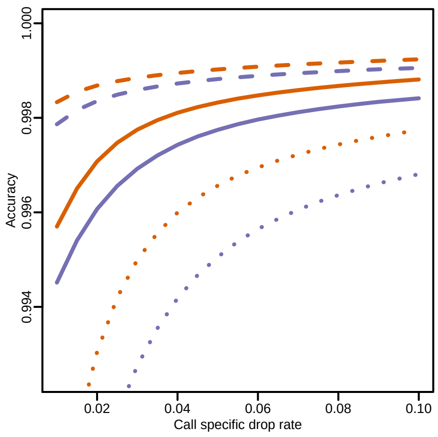

SUPPLEMENTAL FIGURE LEGENDS

Supplemental Figure 1: Accuracy versus dropped rate plots (ADPs) comparing CRLMM and BRLMM for each HapMap dataset are shown in separate plots. The curves are further stratified between heterozygotes and homozygotes as determined by the HapMap gold standard. A) 269 Affymetrix high-quality XBA arrays. B) 269 Affymetrix high-quality HIND arrays. C) 270 Affymetrix high-quality STY arrays. D) 152 Broad Institute first-pass STY arrays. E) 95 Affymetrix first-pass STY arrays. F) 270 Affymetrix high-quality NSP arrays. G) 152 Broad Institute first-pass NSP arrays. H) 95 Affymetrix first-pass NSP arrays.

Supplemental Figure 2: Confidence measure versus accuracy plots (CAPs) comparing CRLMM and BRLMM for each HapMap dataset are shown in separate plots. The curves are further stratified between heterozygotes and homozygotes as determined by the HapMap gold standard. A) 269 Affymetrix high-quality XBA arrays. B) 269 Affymetrix high-quality HIND arrays. C) 270 Affymetrix high-quality STY arrays. D) 152 Broad Institute first-pass STY arrays. E) 95 Affymetrix first-pass STY arrays. F) 270 Affymetrix high-quality NSP arrays. G) 152 Broad Institute first-pass NSP arrays. H) 95 Affymetrix first-pass NSP arrays.

Supplemental Figure 3: The drop rate versus confidence measure are shown for CRLMM and BRLMM. The curves are further stratified between heterozygotes and homozygotes as determined by the HapMap gold standard. A) 269 Affymetrix high-quality XBA arrays. B) 269 Affymetrix high-quality HIND arrays. C) 270 Affymetrix high-quality STY arrays. D) 152 Broad Institute first-pass STY arrays. E) 95 Affymetrix first-pass STY arrays. F) 270 Affymetrix high-quality NSP arrays. G) 152 Broad Institute first-pass NSP arrays. H) 95 Affymetrix first-pass NSP arrays.

Supplemental Figure 4: Validity of drop rates using called data. Drop rates stratified by homozygote and heterozygote calls are plotted against the non-stratified drop rates. The stratification is performed with HapMap gold standard calls (lines) and algorithm

derived calls (triangles and crosses). That the curves derived from HapMap and algorithm results are by and large equivalent demonstrates call rates for homozygotes and heterozygotes from algorithms are valid. Thus in the end, by also using the confidence metric of CRLMM, one can achieve a data set in which all calls are both above a pre-specified accuracy threshold and have equivalent drop rates between homozygotes and heterozygotes. A) 269 Affymetrix high-quality XBA arrays. B) 269 Affymetrix high-quality HIND arrays. C) 270 Affymetrix high-quality STY arrays. D) 152 Broad Institute first-pass STY arrays. E) 95 Affymetrix first-pass STY arrays. F) 270 Affymetrix high-quality NSP arrays. G) 152 Broad Institute first-pass NSP arrays. H) 95 Affymetrix first-pass NSP arrays.

Supplemental Figure 5: ADPs are plotted as in Supplemental Figure 1 except that the drop rate is forced to be the same for both heterozygous and homozygous calls. A) 269 Affymetrix high-quality XBA arrays. B) 269 Affymetrix high-quality HIND arrays. C) 270 Affymetrix high-quality STY arrays. D) 152 Broad Institute first-pass STY arrays. E) 95 Affymetrix first-pass STY arrays. F) 270 Affymetrix high-quality NSP arrays. G) 152 Broad Institute first-pass NSP arrays. H) 95 Affymetrix first-pass NSP arrays.
